# A sensitive one-step real-time PCR for detection of avian influenza viruses using a MGB probe and an internal positive control

**DOI:** 10.1186/1471-2334-6-87

**Published:** 2006-05-25

**Authors:** Livia Di Trani, Barbara Bedini, Isabella Donatelli, Laura Campitelli, Barbara Chiappini, Maria Alessandra De Marco, Mauro Delogu, Canio Buonavoglia, Gabriele Vaccari

**Affiliations:** 1 Department of Food Safety and Veterinary Public Health, Istituto Superiore di Sanità; V.le Regina Elena 299, 00161 Rome, Italy; 2 Department of Parasitic, Immune-Mediated and Infectious Diseases, Istituto Superiore di Sanità; V.le Regina Elena 299,00161 Rome, Italy; 3 National Wildlife Institute, Italy; Via Ca' Fornacetta 9, 40064 Ozzano Emilia (BO) Italy; 4 Department of Veterinary Public Health and Animal Pathology, University of Bologna,; Via Tolara di Sopra 50, 40064 Ozzano Emilia (BO), Italy; 5 Department of Animal Health and Well-Being, Faculty of Veterinary Medicine of Bari, Prov. Per Casamassima Valenzano,70010 Bari, Italy

## Abstract

**Background:**

Avian influenza viruses (AIVs) are endemic in wild birds and their introduction and conversion to highly pathogenic avian influenza virus in domestic poultry is a cause of serious economic losses as well as a risk for potential transmission to humans. The ability to rapidly recognise AIVs in biological specimens is critical for limiting further spread of the disease in poultry. The advent of molecular methods such as real time polymerase chain reaction has allowed improvement of detection methods currently used in laboratories, although not all of these methods include an Internal Positive Control (IPC) to monitor for false negative results.

Therefore we developed a one-step reverse transcription real time PCR (RRT-PCR) with a Minor Groove Binder (MGB) probe for the detection of different subtypes of AIVs. This technique also includes an IPC.

**Methods:**

RRT-PCR was developed using an improved TaqMan technology with a MGB probe to detect AI from reference viruses. Primers and probe were designed based on the matrix gene sequences from most animal and human A influenza virus subtypes. The specificity of RRT-PCR was assessed by detecting influenza A virus isolates belonging to subtypes from H1–H13 isolated in avian, human, swine and equine hosts. The analytical sensitivity of the RRT-PCR assay was determined using serial dilutions of *in vitro *transcribed matrix gene RNA. The use of a rodent RNA as an IPC in order not to reduce the efficiency of the assay was adopted.

**Results:**

The RRT-PCR assay is capable to detect all tested influenza A viruses. The detection limit of the assay was shown to be between 5 and 50 RNA copies per reaction and the standard curve demonstrated a linear range from 5 to 5 × 10^8 ^copies as well as excellent reproducibility. The analytical sensitivity of the assay is 10–100 times higher than conventional RT-PCR.

**Conclusion:**

The high sensitivity, rapidity, reproducibility and specificity of the AIV RRT-PCR with the use of IPC to monitor for false negative results can make this method suitable for diagnosis and for the evaluation of viral load in field specimens.

## Background

Influenza A viruses belong to *Orthomyxoviridae*, a family of enveloped negative-sense, segmented single stranded RNA viruses. Based on major differences within the hemagglutinin (HA) and neuraminidase (NA) proteins, 16 HA and 9 NA subtypes have been recognized, all of which have been isolated from avian species [1-3]. Only the H1, H2, and H3 subtypes have established stable humans lineages, whereas H5, H7 and H9 subtypes have been detected sporadically in humans. Among avian species, wild aquatic birds represent the natural reservoir of all influenza A subtypes from which influenza viruses are introduced in to all other species affected in nature, including domestic poultry. Infections of domestic avian species with low pathogenic avian influenza (LPAI) viruses can be asymptomatic or cause a wide range of clinical signs varying from mild respiratory disease to more severe diseases affecting the respiratory and enteric systems. Highly pathogenic avian influenza viruses (HPAI) cause rapid mortality in poultry, which often approaches 100% of incidence [4]. There is evidence that LPAI viruses carried by wild birds can acquire high virulence after direct transmission to and replication in domestic species. Genetic reassortment of avian, swine and human influenza viruses was considered, until the 1990s, to be the main mechanism whereby novel viruses containing animal virus genes could transmit to humans. However, the zoonotic events in Asia and in Europe caused by H5N1, H9N2 and H7N7 subtypes respectively indicate that AIVs are capable of infecting humans directly [5]. The link between human and avian influenza has raised concern among public health authorities and the scientific community about the prevalence and pandemic potential of AIVs.

Prompt identification of AIVs circulating in the field can help control viral spread in poultry, thereby reducing the potentially serious economic damage as well as the exposure of humans to AIVs. Therefore rapid, highly specific and sensitive assays are required in avian influenza virus diagnosis.

Methods in routine use for avian influenza virus detection and characterization include virus isolation in embryonated eggs followed by identification by haemagglutination-inhibition (HI) or ELISA tests. Viral culture assay is quite sensitive, but time consuming and technically demanding, and requires the presence of infectious viral particles; instead, ELISA for antibodies or antigen is a test of limited specificity [6]. An approach for the rapid identification and detection of AIVs is the application of reverse transcription-PCR (RT-PCR) [7]. Although PCR is one of the most sensitive and specific techniques and the presence of infectious virus is not necessary, the assay requires multiple manipulations of the samples after the amplification step, thus increasing the risk of carryover contamination, and usually does not include an Internal Positive Control (IPC).

Recently, quantitative, fluorescence-based real-time PCR assays have been developed in different formats [8-10]. These methods exhibit good sensitivity, broad dynamic range and are capable of detecting all AIVs subtypes. However, most of these assays detect influenza virus RNA in the absence of an internal control; the inclusion of an amplification control is particularly useful to monitor for false negative results due to RNA degradation or to inhibitory factors, potentially present in clinical samples [11]. This feature can be of critical importance in avian influenza diagnosis: failure to identify a positive AI outbreak in a poultry farm, particularly in the case of highly pathogenic viruses, would not allow to adopt promptly the necessary actions for eradication purpose, thus favouring the undetected spread of the virus to other flocks. This failure could have even more serious consequences in the case of domestic ducks, since in these birds infections with highly pathogenic viruses, such as the H5N1 strains from South East Asia, are often asymptomatic [4].

RRT-PCR assays with new Minor Groove Binder (MGB) probes offer several advantages over existing probes [12]. In fact, a DNA probe with conjugated MGB groups forms a stable duplex with a single-stranded DNA target; therefore it can be shorter than those without MGB groups. In addition the presence of a 3' dark quencher reduces the background fluorescence. Both this features have been shown to improve the specificity and sensitivity of MGB RRT-PCR assay in comparison to RRT-PCR tests using unmodified DNA probes [12].

In this paper, a sensitive MGB RRT-PCR was developed for detection of AIVs with the inclusion of an IPC in order to monitor for possible failures in the diagnostic evaluation of field samples.

## Methods

### Viruses

Human influenza type A and type B viruses and a number of avian viral pathogens used to test the specificity of influenza A matrix and IPC assays are listed in Table 1 and were selected from the repository of Istituto Superiore Sanità laboratories.

**Table 1:** List of influenza A viruses isolates and other pathogens tested for RRT-PCR specificity assay.

**ISOLATE**	**HA SUBTYPE**	**RESULT**
A/Mallard/Italy/70/96	H1N1	positive
A/Mallard/Italy/35/99	H2N3	positive
A/Duck/Ukraine/63	H3N8	positive
A/Mallard/Italy/616/01	H4N6	positive
A/Chicken/Italy/312/97	H5N2	positive
A/Mallard/Italy/80/93	H5N2	positive
A/Chicken/Italy/9097/97	H5N9	positive
A/Mallard/Italy/41/00	H6N8	positive
A/Turkey/Italy/214845/02	H7N3	positive
A/Turkey/Ontario/618/68	H8N4	positive
A/Turkey/Wiss/66	H9N2	positive
A/Coot/Italy/125/94	H10N8	positive
A/Mallard/Italy/243/00	H11N6	positive
A/Duck/Alberta/60/76	H12N5	positive
A/Gull/Maryland/704/77	H13N6	positive
A/Equine/New Market/2/93	H3N8	positive
A/Equine/Roma/1/91	H3N8	positive
A/Swine/Italy/1421/95	H1N1	positive
A/Swine/1184/92	H3N2	positive
A/New Caledonia/20/99	H1N1	positive
A/Roma/3/03	H3N2	positive
B/Guandong/120/00	NA	negative
B/Yamagata/16/88	NA	negative
PMV 2 (Ck/Ca/Yucaipa/56)	NA	negative
PMV 3 (Tk/1087/82)	NA	negative
PMV4 (Dk/HK/D3/75)	NA	negative
TRTV (But 1FF 8544)	NA	negative
IBDV (D-78)	NA	negative
IBV (M41)	NA	negative

NA: not applicable

PMV: paramixovirus

TRTV:turkey rhinotracheitis virus

IBDV:infectious bursal disease virus

IBV:avian infection bronchitis virus

A/Turkey/Italy/214845/02 (Ty/214845) H7N3 virus [13] was propagated in the allantoic cavities of 11-day-old embryonated chicken eggs to produce working stocks of the virus for use as reference virus strain for assay standardization. Virus stocks of Ty/214845 were titrated by end point dilution in Madin Darby canine kidney (MDCK) cells, and the 50% tissue culture infectious doses (TCID_50_) were calculated as previously described [14] in a modified ELISA test that identifies the expression of influenza A virus nucleoprotein in infected cells. Moreover to determine the virus titer in 50% egg infectious dose (EID_50_), 10-day old fertile hen eggs were inoculated with Ty/214845 and the allantoic fluid was collected and tested for hemagglutination. The virus titer was determined using the Reed and Muench method [15]. The Ty/214845 virus, with a TCID_50 _of 10^7.34^/ml and a EID_50 _of 10^8.25^/ml, was used as the stock virus in the assay. A series of 10-fold dilutions of the stock virus sample in transport medium were prepared and stored at -80°C until use for RNA extraction. Transport medium consisted of PBS/Glycerol (1:1) supplemented with potassium penicillin (1,000 U/ml), streptomycin sulfate (200 μg/ml), gentamicin sulfate (240 μg/ml), polymyxin B (100 Unit/ml) and mycostatin (50 Unit/ml). These antibiotics were from Sigma-Aldrich Co. (St Louis, MI).

### Field specimens

To evaluate the applicability of the test as a diagnostic method in the screening of field specimens, we analysed retrospectively one hundred samples collected during surveillance studies in wild waterfowl [16]. Cloacal swabs were collected from ducks using sterile cotton swabs and resuspended in the transport medium described above. Ten of these samples had been shown to be positive for influenza type A by standard virus isolation procedures. Twenty pools of five cloacal swabs each were prepared before viral RNA extraction. Ten of the pools were obtained mixing one positive with four negative AI samples, while each of the remaining pools contained five negative AI cloacal swabs only. All twenty pools were spiked with IPC RNA before the extraction step (see section Internal Positive Control).

### Primer and probe design

The primers and probe were designed based on the sequence homology of the matrix gene (segment 7) among different subtypes of influenza A strains of avian, human, swine and equine origin. A 147 bp region located at the 5'end of the matrix gene (nucleotides 32–179), which represents one of the highly conserved regions of the virus gene, was chosen as the target region for primer amplification. The fluorogenic MGB probe, labelled at 5' with FAM and at 3' with a dark quencher dye, was designed to anneal to an internal sequence of the amplified region. The sense and antisense primers and MGB probe were designated M-Flu1, M-Flu2, and M-Fluprob (Table 2), respectively, and were designed using the Primer Express v. 1.5 software (Applied Biosystems, Foster City, CA).

**Table 2:** Primers and MGB-probe designed in this work

**NAME**	**SEQUENCE (5'-3')**	**LOCATION (nt)**	**SENSE**
M-Flu1	CTTCTAACCGAGGTCGAAACGTA	32–54	+
M-Flu2	GGATTGGTCTTGTCTTTAGCCA	158–179	-
M-Fluprob	FAM-CTCGGCTTTGAGGGGGCCTGA-MGB	74–94	-

### Standard RNA synthesis

*In vitro *transcribed matrix gene RNA of Ty/214845 was used to determine the detection limit of the assay as a positive control. The entire 1027 bp long matrix gene was amplified with a specific set of primers (sequences are available upon request) and the product was purified using the QIAquick gel extraction kit (Qiagen) and ligated to the PCR2.1 vector using the TOPO™ TA cloning kit (Invitrogen Corp, Carlsbad, CA). To verify that the construct obtained (pFluA) had no mutations in the sequences corresponding to the primers and probe positions and to determine the sequence and orientation of the insert, nucleotide sequencing was performed using ABI BigDye Terminator v. 1.1 sequencing kit and the ABI-Prism 310 sequencer (Applied Biosystems). The plasmid pFluA, which possesses a T7 promoter, was linearised at the end of the matrix protein gene and then purified using the Wizard DNA Clean-up kit (Promega, Madison, WI). DNA concentration at two dilutions (1:100 and 1:1000 in TE buffer, pH 8) was measured as OD units at 260 nm and the number of plasmid copies in the extract was calculated from the molecular weight of the plasmid and the insert.

Ten μg of linearized plasmid was transcribed using RiboMax kit from the T7 promoter according to the manufacturer's instructions and quantified by spectrophotometer analysis RNA copy number was then determined following the method of Fronhoffs [17].

Ten-fold dilutions of the RNA transcript, ranging from 1 to 10^8 ^copies/μl, were prepared in sterile water and used to determine the analytical sensitivity of the RRT-PCR assay.

### Internal positive control

The rodent glyceraldehyde-3-phosphate dehydrogenase (GAPDH) RNA was chosen as an IPC. The commercial kit (supplied by Applied Biosystems) contained GAPDH reagents designed to detect rat, mouse and chinese hamster GAPDH genes by means of gene specific primers and probe.

For evaluation of inhibition effects, 25, 2.5, 0.25, 0.025, 0.0025 0.00025 ng of the IPC RNA were added to the RRT-PCR in addiction to 5 copies of *in vitro *transcribed matrix gene RNA. In the evaluation of the extraction phase with the reference virus, 0.3 ng of RNA IPC were added prior to this step in order to obtain in the final RRT-PCR a maximum concentration of 0.025 ng. This was calculated taking into account that each sample is eluted in 60 μl as above described and only 5 μl of this mixture is used in the RRT-PCR.

The GAPDH RNA was detected using forward and reverse primers and the TaqMan VIC labelled probe, according to the instructions of the manufacturer, in the multiplexed RRT-PCR assay described below.

### Extraction of viral RNA

Total RNA was extracted from all types of viruses using QiAmp Viral RNA Mini kit (Qiagen GmbH, Hilden, Germany) according to the manufacturer's instructions. Briefly, 210 μl of sample containing transport medium spiked with 6 μl of IPC RNA were mixed with the provided lysis buffer and left for at least 10 min at room temperature. After the addition of 560 μl of 97% ethanol, the liquid was repeatedly run in a spin-column more than once until it was finished. Finally, RNA was eluted in 60 μl of RNase-free water and, after addition of 20 unit of RNase inhibitor, was stored at -80°C until use. In the negative control, sterile water was added instead of the specimen.

Influenza A viruses, listed in Table 1, were also extracted without IPC RNA, in order to test the rodent primers and probe specificity.

### One-Step Real-Time RT-PCR

RNA from all samples, were amplified by RRT-PCR assay, run in an ABI Prism 7000 SDS Real-Time apparatus (Applied Biosystems) using the Superscript III Platinum One-step qRT-PCR kit (Invitrogen). The 25 μl reaction volume contained 5 μl of extracted RNA, 1X Superscript III Platinum One-step qRT-PCR reaction mix, 0.5 μl of ROX reference dye as a passive reference, 0.2 μM of each probe and 0.4 μM of each one of the primers for both the matrix gene and rodent RNA. The following thermal profile was used: a single cycle of reverse transcription for 30 min at 45°C, 2 min at 95°C for reverse transcriptase inactivation and DNA polymerase activation followed by 40 amplification cycles of 15 sec at 95°C and 1 min at 60°C each (annealing-extension step). Triplicate negative and positive controls were included in each experiment. Each fluorescent reporter signal was measured against the internal reference dye (ROX) signal to normalize for non-PCR-related fluorescence fluctuations between samples. The data were collected at the annealing step of each cycle and the threshold cycle (Ct) for each sample was calculated by determining the point at which the fluorescence exceeded the threshold limit.

The standard curve was calculated automatically by plotting the Ct values against each standard of known concentration and by extrapolating the linear regression line of this curve.

### Specificity and analytical sensitivity of RRT-PCR

Primers and probe specificity for type A influenza viruses and for IPC were assessed by testing amplification of RNA from influenza virus isolates belonging to the most commonly isolated HA subtypes (H1–H13), represented by either Eurasian or North American lineage avian strains, human, equine and swine viruses, as well as type B human influenza viruses and a panel of avian viral pathogens (Table 1).

To test the analytical sensitivity of the RRT-PCR assay, RNA extracted from ten-fold diluted samples of Ty/214845, containing 1 to 0,0001 TCID_50 _and 80 to 0.008 EID_50 _of virus were tested. To determine the detection limit of the assay in terms of matrix gene copies number, serial dilutions of *in vitro *transcribed RNA, ranging from 5 × 10^8 ^to 5 copies/reaction were analysed. The detection limit of the assay was determined as the last dilution at which all 10 replicates of each dilution gave a positive result.

### Reproducibility of RRT-PCR

To calculate the reproducibility of the test, three different concentrations of the Ty/214845 virus, corresponding to 100, 1 and 0,01 TCID_50 _respectively, were submitted to absolute quantification using the standard curve built with *in vitro *transcribed RNA (see Standard RNA synthesis section). Each dilution was quantified in triplicate. The Coefficient of Variation (CV) for evaluation of the intra-assay repeatability was calculated by testing the three dilutions ten times in the same experiment. To estimate the inter-assay reproducibility, the three dilutions of Ty/214845 virus were analyzed in tests independently run in different days.

### Conventional RT-PCR for detection of avian influenza viruses

Conventional RT-PCR for influenza A virus, which amplifies a 244 bp fragment of the matrix gene coding sequence, was also performed as previously described [7] on influenza A viruses (Table 1) and clinical specimens.

## Results

### Specificity

The primer and probe set was capable of detecting all type A influenza viruses, whereas no signal amplification was observed with influenza B viruses and other avian pathogens. Moreover all samples, including the negative control for the amplification of the matrix gene, gave a positive result in the RRT-PCR assay for IPC specific amplification.

In the IPC specificity assay no signal of fluorescence was obtained using primers and probe specific for rodent RNA amplification.

### Analytical sensitivity

**Figure 1:**
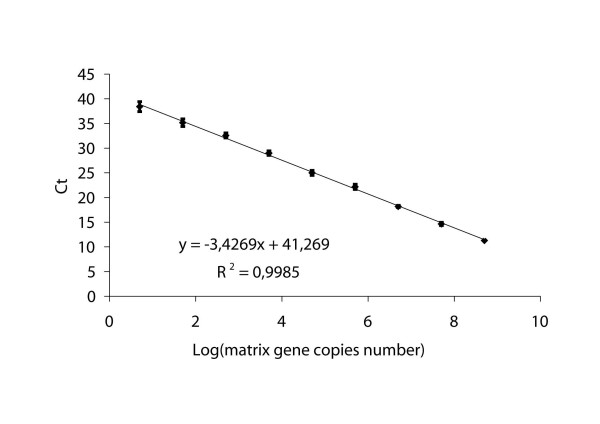
Standard curve of the matrix gene real-time RRT-PCR assay. Serial 10-fold dilutions of *in vitro *transcribed matrix RNA standard (from 1 to 10 ^8 ^copies/μl) were plotted against the threshold cycle. Each plot represents the mean of 10 replicate amplifications of each dilution. The coefficient of determination (R^2^) and the equation of the regression curve (y) calculated.

The analytical sensitivity of the influenza A RRT-PCR assay was determined by amplification of both RNA extracted from dilutions of a TCID_50 _and EID_50 _titrated stock of reference virus and of *in vitro *transcribed matrix RNA. The RRT-PCR assay could detect up to 0.001 TCID_50 _of the Ty/214845 reference virus (equivalent to 0.08 EID_50_) as few as 5 to 50 RNA matrix gene copies per reaction. Evaluation of the assay analytical sensitivity was performed in three different runs. Figure 1 presents one of the experiments that indicates a linear correlation between the Log of the matrix gene copy number and the Ct, with a regression line showing a slope of 3.43 (R^2 ^= 0.998). To compare the analytical sensitivity of RRT-PCR and RT-PCR, serial dilutions of known amounts of *in vitro *transcribed RNA were also tested by conventional RT-PCR. The results demonstrated that the RT-PCR could detect up to 5 × 10^2 ^copies of RNA/reaction, which represents a 10-100-fold higher detection limit than that observed with RRT-PCR.

### Reproducibility

The intra and inter-assay Coefficients of Variation were calculated using three dilutions of the Ty/214845 virus, corresponding to 100, 1 and 0,01 TCID_50_, in order to simulate positive samples with a wide range of concentrations. The CV intra-assay for each dilution was 25, 27 and 30% respectively, whereas the CV inter-assay was 27, 30, and 41% respectively.

### Internal Positive Control and interpretation of results

The decision to include an IPC in the final assay, added to the matrix gene assay, prompted us to consider the influence of such additional amplification on the detection limit for the influenza virus target and the specificity of the assay to detect the IPC. The highest amount of IPC RNA, which did not influence the Ct for matrix gene RNA amplification, was equal to 0.025 ng/reaction. This amount was then added to the sample containing the lowest amplified quantity of reference virus, corresponding to 0.001 TCID_50 _prior to the extraction step. The amplification of matrix gene RNA confirmed that the detection limit of the reference virus was not influenced by the previously established amount of IPC.

### Detection of viral RNA in field samples

To assess whether the RRT-PCR assay allows the detection of viral RNA in biological samples collected in the field, twenty pools of cloacal swabs, obtained from one hundred faecal samples collected from wild birds, were tested by RRT-PCR. The assay detected the presence of influenza A virus RNA in ten pools, previously found to be positive by both virus culture and conventional RT-PCR (data not shown). For the remaining ten pools, a positive signal was obtained only for rodent RNA, indicating the absence of false negative results.

## Discussion

The epidemics of avian influenza in Asia and, more recently, in some European regions [18], have caused considerable public concern and raised the need of continued vigilance for rapid virus detection in poultry. Rapid and sensitive influenza diagnosis in domestic birds is fundamental to allow the implementation of control measures aimed at containing the outbreaks in poultry, reducing human exposure. Moreover, early influenza detection is important for the screening of potential carriers of influenza A such as wild birds, whose role in the H5N1 spread throughout Asia and most European countries has been hypothesised [18].

Methods used for influenza A identification in birds should be specific enough to allow detection of antigenically and genetically different influenza subtypes. Among them, the RT-PCR technique is widely used to detect influenza viruses directly in specimens collected from animal species susceptible to influenza virus infection and from humans [7].

Recently, new molecular approaches have been described, involving specific fluorogenic probes that allow the simultaneous amplification and visualization of the viral nucleic acid in real-time. Indeed, RRT-PCR assays improve the sensitivity and specificity of gene detection, reduce significantly hands-on time, and allow quantitation of the total amount of nucleic acids.

In this study we present data on a highly sensitive assay for AIV detection by real-time RT-PCR, based on the new MGB probe technology, which includes the use of an IPC to monitor for false negative results. Moreover, our assay could be easily adapted in a quantitative format by using a previously quantified RNA to create a standard curve to which results from unknown samples can be compared.

For this purpose, we designed primers and a probe specific for a region of the matrix gene that is strictly conserved for most influenza A sequences available. In this way, we expected to be able to detect influenza A viruses belonging to all subtypes, and lineages within subtypes. The collected results showed that our RRT-PCR assay is highly specific for detection of all tested influenza A virus strains.

A novel MGB probe for matrix gene detection was used; this type of probe has melting temperatures higher than the common Taq-Man probes, thus allowing the hybridization to the target sequence and consequently the generation of fluorescence signals, even also in the presence of possible mutations within this highly conserved region. Although single-step RRT-PCR is reported to be less sensitive than a two-step amplification method [19], the use of a one-step RRT-PCR was performed in this study to prevent the risk of cross contamination and to increase the speed of the test. Moreover, the proposed RRT-PCR assay was 10-fold more sensitive compared to the RRT-PCR already published [9,10] and 10–100 fold more sensitive than conventional RT-PCR. This is extremely important in routine diagnostic studies particularly when the amount of influenza A virus RNA in field specimens is low.

Multiple negative controls as well as positive controls should be included in diagnostic RRT-PCR in order to achieve an acceptable level of confidence for the absence of false-positive and/or false-negative results. However, since such controls are generally run in separate tubes, no information about the performance of the extraction and amplification reaction in the sample-containing tubes is usually available. In particular, if amplification is partly inhibited or if there is a partial loss of nucleic acid during sample processing, the sample Ct of IPC will be higher than under ideal conditions, and will consequently yield an artificially low RNA reading on the standard curve [11]. To circumvent this problem, in our system an IPC was added to each sample before the extraction step; it consists of a second target sequence, represented by a rodent RNA, unrelated to the sequence to be detected and available in a commercial kit. Adding the IPC before influenza RNA isolation allows monitoring of the whole process from extraction to RRT-PCR [20,21]. Furthermore, this IPC can be used in multiplex RRT-PCR for detection of other pathogens. Important problems in multiplex RRT-PCR assays with an IPC are competitive effects and loss of sensitivity [20,21] that could be avoided by using low concentrations of the IPC. Nevertheless, an inhibition of IPC amplification could be observed also when very high amounts of RNA target were present. When this partial or complete inhibition of IPC detection caused by high amounts of target RNA occur, it is not significant since IPC is used to monitor for false negative results in the presence of low levels of target RNA [20]. The choice of an RNA as internal positive control was made considering the public availability of such reagents, and represents a potential possible first step in the harmonization of the RRT-PCR assay for influenza diagnostic.

The test described is extremely sensitive, being able to detect 5 to 50 gene copies/reaction of *in vitro *transcribed RNA. The minimum detectable amount of AI reference virus corresponded to 0.001 TCID_50_/reaction and 0.08 EID_50_/reaction respectively.

Detection limits for the influenza A virus matrix gene assessed in other TaqMan-PCR assays ranged from 0.006 to 0.2 TCID_50_/ml [22,23,7]. Nevertheless, a comparison of the sensitivity between the different formats of RRT-PCR is difficult because of the use of different viral strains, viral concentration methods and lack of IPC. The availability of reference material is of paramount importance to compare the sensitivity of different diagnostic assays particularly in the light of the current avian influenza H5N1 crisis which would require a global approach in diagnostic laboratories [24]. Finally, results indicate that our test has a good reproducibility, as shown by a low CV within and between performed assay.

The suitability of the RRT-PCR test described in this study as a diagnostic tool for AIV detection was confirmed by testing samples taken from naturally infected birds. In comparison with conventional RT-PCR, the number of viral genome molecules of standard RNA detected by RRT-PCR assay was found to be 2 log lower in these specimens.

## Conclusion

The MGB assay described here could be used for the detection of all subtypes of influenza A viruses tested. Quantification of viral load could be important in the study of viral replication both in cell culture models and in animals experimentally infected. This quantification could provides useful tool to evaluate the efficacy of new vaccines and antivirals against AI [25]. This assay, with the insertion of standard curves or a calibrator sample in each run could be easily adapted in a quantitative format.

RRT-PCR tests for avian influenza virus detection have been developed in different formats [26,9,10]. Most of them recognise highly conserved sequences within type-specific genes, such as that coding for the matrix gene, alone or in combination with primers and probe specific against the H5 and/or H7 hemagglutinin subtypes, those capable of evolving into a highly pathogenic phenotype.

The inclusion of the IPC in the assay represents an improvement in the design of a RRT-PCR; IPC is useful to monitor for false negative results due to PCR failure caused by expired reagents, poor technique, equipment failure or presence of enzyme inhibitors in biological samples. Moreover the possibility of screening a large number of samples in a rapid, sensitive and reproducible way could make this assay a possible suitable tool for the routine diagnostic laboratories possessing a RRT-PCR equipment. Infact, although the analysis of a small number of field samples has been reported, this is a first step in the field validation of our RRT-PCR assay and more extensive work, involving the application of the assay on field samples, including also specimens from poultry, will be necessary in the future.

## Competing interests

The author(s) declare that they have no competing interests.

## Authors' contributions

LDT conceived and designed the study and wrote first draft of manuscript, BB carried out the molecular tests and participated in the design of the study and in the critical review of the manuscript, ID provided reference viruses and applied for funding, LC participated in the design of the study and provided expert input for writing and reviewing of the manuscript, BC carried out the assay development and participated in the standard RNA synthesis, MDM and MD provided field specimens, CB provided expert input for writing and critical review of the manuscript, GV have contributed in the assay design, performed data analysis and supervised the study. All authors have read and approved the final manuscript.

## Pre-publication history

The pre-publication history for this paper can be accessed here:


